# Skeletal stem cells: a game changer of skeletal biology and regenerative medicine?

**DOI:** 10.1093/lifemedi/lnac038

**Published:** 2022-09-14

**Authors:** Qiaoling Zhu, Lei Ding, Rui Yue

**Affiliations:** Institute for Regenerative Medicine, Shanghai East Hospital, Frontier Science Center for Stem Cell Research, Shanghai Key Laboratory of Signaling and Disease Research, School of Life Sciences and Technology, Tongji University, Shanghai 200092, China; Columbia Stem Cell Initiative, Department of Rehabilitation and Regenerative Medicine and Department of Microbiology and Immunology, Columbia University Irving Medical Center, New York, NY 10032, USA; Institute for Regenerative Medicine, Shanghai East Hospital, Frontier Science Center for Stem Cell Research, Shanghai Key Laboratory of Signaling and Disease Research, School of Life Sciences and Technology, Tongji University, Shanghai 200092, China; Shanghai Institute of Stem Cell Research and Clinical Translation, Shanghai 200120, China

**Keywords:** skeletal stem cells, hematopoietic stem cells, bone marrow microenvironment, skeletogenesis, regenerative medicine

## Abstract

Skeletal stem cells (SSCs) were originally discovered in the bone marrow stroma. They are capable of self-renewal and multilineage differentiation into osteoblasts, chondrocytes, adipocytes, and stromal cells. Importantly, these bone marrow SSCs localize in the perivascular region and highly express hematopoietic growth factors to create the hematopoietic stem cell (HSC) niche. Thus, bone marrow SSCs play pivotal roles in orchestrating osteogenesis and hematopoiesis. Besides the bone marrow, recent studies have uncovered diverse SSC populations in the growth plate, perichondrium, periosteum, and calvarial suture at different developmental stages, which exhibit distinct differentiation potential under homeostatic and stress conditions. Therefore, the current consensus is that a panel of region-specific SSCs collaborate to regulate skeletal development, maintenance, and regeneration. Here, we will summarize recent advances of SSCs in long bones and calvaria, with a special emphasis on the evolving concept and methodology in the field. We will also look into the future of this fascinating research area that may ultimately lead to effective treatment of skeletal disorders.

## Introduction

Although long considered as body scaffolds to protect internal organs and enable locomotion, the skeleton has been increasingly recognized with versatile functions, such as calcium/phosphate reservoir, endocrine organ, and hematopoietic microenvironment [1–6]. The skeleton is also the home to two distinct types of tissue-specific stem cells, namely, skeletal stem cell (SSCs) and hematopoietic stem cell (HSCs), which maintain the homeostasis and regeneration of the skeletal and hematopoietic systems in adults, respectively. SSCs were originally found in the bone marrow stroma and for decades has been called several interchangeably terms, including bone marrow osteogenic stem cells/stromal stem cells [7, 8], mesenchymal stem cells (MSCs) [9], and multipotent bone marrow stromal cells (BMSCs) [10]. “Bone marrow SSCs” seems to be a more appropriate name for these cells, because it specifies their tissue origin and distinguishes them from recently discovered SSC populations in the growth plate, perichondrium, periosteum, and calvarial sutures. Current consensus is that skeletal development, maintenance, and regeneration are regulated by the concerted action of different waves of SSCs, which are spatially and functionally distinct. Reliable phenotypic markers and robust functional assessments of SSCs have just been established. With these tools, the development of SSCs and their functional changes in aging and degenerative diseases are the focus of current investigations. In this review, we will focus on these recent advances of SSC biology, and shed light on the future application of SSCs in regenerative medicine.

## Endochondral and intramembranous ossification

**Figure 1.**
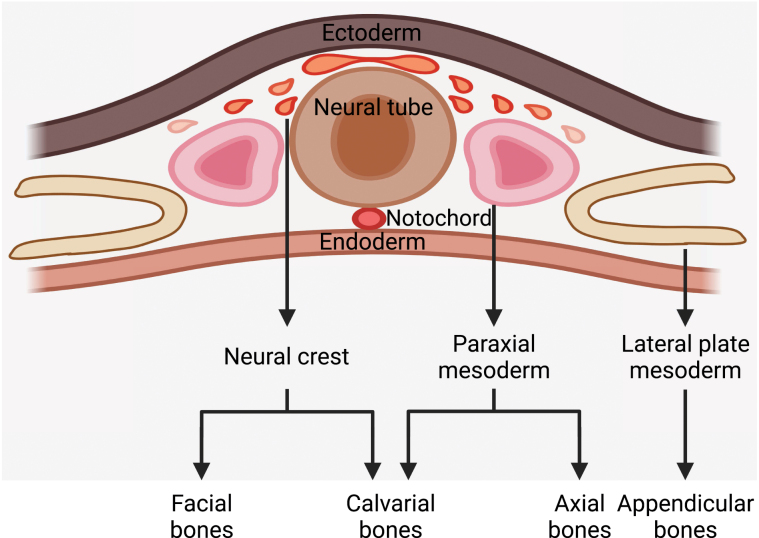
Embryonic origins of the skeleton. The appendicular bones, such as limb bones, are derived from lateral plate mesoderm. The axial bones, such as the vertebrae, are derived from paraxial mesoderm. The facial bones are mainly derived from cranial neural crest. The calvarial bones are derived from both cranial neural crest and paraxial mesoderm.

Different parts of the skeleton have distinct embryonic origins [11]. Limb bones and vertebrae are derived from lateral plate mesoderm and paraxial mesoderm [12, 13], respectively, via endochondral ossification. In contrast, calvarial bones are derived from both neural crest and paraxial mesoderm [14, 15] via intramembranous ossification (Fig. 1).

Endochondral ossification is characterized by the formation of a cartilaginous template before the onset of ossification. The development of limb bones, e.g. begins with the condensation of limb bud mesenchymal progenitor cells, which generate the hyaline cartilage primordium [16]. Following rapid elongation, chondrocytes at the center of cartilage primordium exit cell cycle and undergo hypertrophy. The primordium is subsequently invaded by perichondrial skeletal progenitors along with the blood vessels to form the primitive bone marrow cavity, or primary ossification center (POC) [17]. Ossification then takes place by either osteogenic differentiation of the invaded skeletal progenitors on top of the cartilage matrix [18, 19], or transdifferentiation of the hypertrophic chondrocytes into osteoblasts/osteocytes [20–22]. Similar invasion events take place again at both ends of the long bones shortly after birth, leading to the formation of secondary ossification center that separate the growth plate from articular cartilage. The growth plate chondrocytes keep proliferating to maintain longitudinal growth of the long bones until mineralized in adulthood, when the long bones reach maximal length and stop growing.

In stark contrast, some flat bones, such as the calvaria, are mainly formed by intramembranous ossification without the cartilage intermediate [17]. Mesenchymal progenitors derived from either neural crest or paraxial mesoderm condense directly differentiate into osteoblasts, which deposit osteoid matrix to form discrete ossification centers in the calvaria [23–25]. Interestingly, although non-endochondral cartilage, such as the parietal cartilage, transiently exists during calvarial development, it does not undergo hypertrophy and is degraded in an MT1-MMP-dependent manner [26, 27]. The skeletal progenitors generating the calvarial cartilage, as well as their contribution to intramembranous ossification and suture formation, remain to be elucidated.

The molecular mechanisms by which chondrogenesis and osteogenesis are regulated have been extensively studied and reviewed elsewhere [28–32]. Here, we focus on the identification and characterization of the SSC activity within different waves of skeletal progenitors that underlie endochondral and intramembranous ossification.

## Assays in SSC biology

**Figure 2.**
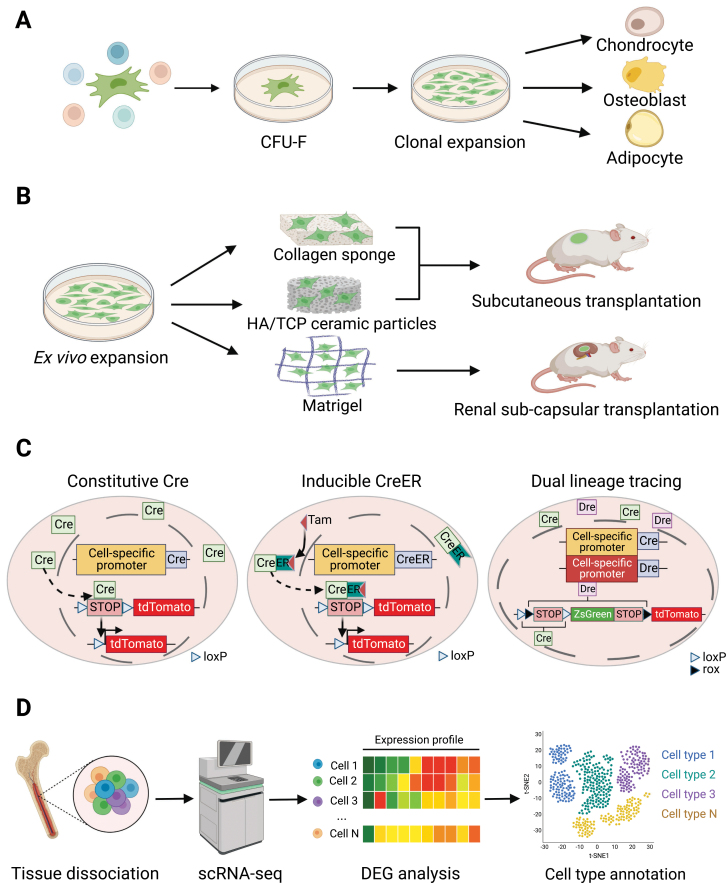
Assays for studying SSCs. (A) CFU-F colony formation. SSCs with clonogenic capacity can be isolated by plastic adherence during *ex vivo* monolayer culture. Single colonies formed by CFU-Fs can be induced to generate adipocytes, osteoblasts, and chondrocytes *in vitro* to test their multilineage differentiation capacities. (B) Ectopic bone formation after *in vivo* transplantation. Collagen sponges are only used for transplantation of mouse cells, while HA/TCP ceramic particles can be used for both mouse and human cells. Notably, none of these methods can generate cartilage. Matrigel can be used for renal subcapsular transplantation of both mouse and human cells, which generate all skeletal lineage cells. Immunodeficient mice should be used when human cells are transplanted. (C) Genetic lineage tracing. For constitutive lineage tracing, the Cre recombinase is driven by a cell-specific promoter. Cre recognizes two loxP sites, and excises the transcriptional termination sequence between them, allowing expression of the reporter gene (tdTomato). Cre-expressing cells and their progeny cells will be permanently labeled. For inducible lineage tracing, upon Tamoxifen (Tam) administration, the CreER recombinase translocates from the cytoplasm into nucleus to excises the transcriptional termination sequence between the two loxP sites, allowing expression of the reporter gene. For dual lineage tracing, orthologous Dre-rox and Cre-loxP recombination systems are used to perform simultaneous or mutually exclusive tracing of Dre^+^ and/or Cre^+^ cells. (D) Single-cell RNA-sequencing. Skeletal tissues are dissociated and subjected to scRNA-seq to resolve cellular heterogeneity and identify potential SSC populations.

Colony-forming unit-fibroblast (CFU-F) culture is a classical *in vitro* assay to detect SSC activity [33] (Fig. 2A). After mechanical or enzymatic dissociation into single cells, SSCs with clonogenic capacity adhere to plastic culture dish to form single colonies, which could be expanded and serially passaged to assess their self-renewal potential. Single colonies formed by CFU-Fs can be differentiated into adipocytes, osteoblasts, and chondrocytes *in vitro* to demonstrate multilineage differentiation potential of SSCs [34] (Fig. 2A). Notably, non-clonal culture of CFU-Fs is inappropriate to detect stem cell activity, as the trilineage differentiation, if any, could be explained by a mixture of three populations of unipotent progenitors.

*In vivo* transplantation to form ectopic bones is another classical assay to study SSCs (Fig. 2B). In this assay, *ex vivo* expanded cells (~10^6^) are loaded onto collagen sponge (for mouse cells only) or hydroxyapatite (HA)/tricalcium phosphate (TCP) ceramic particles (for mouse and human cells), followed by subcutaneous transplantation into recipient mice. Four to eight weeks later, ectopic bones are readily formed, which contain donor-derived osteoblasts/osteocytes and adipocytes, as well as recipient-derived vasculatures and hematopoietic cells [33]. Of note, chondrocytes are hardly generated by this method, possibly because a compact 3D structure is needed for efficient chondrogenesis. Therefore, *in vitro* chondrogenic differentiation by micro-mass or 3D pellets is recommended to independently test the chondrogenic potential [33]. In recent years, renal subcapsular transplantation has become a gold standard for assessing SSC activity [35] (Fig. 2B). In this assay, a relatively small number (10^3^–10^5^) of flow cytometrically sorted primary cells, or *ex vivo* cultured cells, are mixed with Matrigel (2–10 μL), followed by renal subcapsular transplantation [36–41]. Ectopic bones are formed 3–4 weeks after transplantation, with donor-derived osteoblasts, chondrocytes and adipocytes, as well as recipient-derived vasculatures and hematopoietic cells. Secondary transplantation is required to demonstrate the self-renewal capacity of SSCs. The subcutaneous space is another site to perform SSC transplantation [42], which is easily accessible with less technical demands. However, it is less vascularized as compared to subcapsular space of the kidney, which could yield different transplantation results [43, 44]. Immunodeficient recipient mice should be used when studying human SSCs (HSSCs) by either subcutaneous or renal transplantation [45–47].

*Ex vivo* culture or *in vivo* transplantation of SSCs, especially after enzymatic dissociation and flow cytometry sorting, could impose artificial stresses that compromise their stem cell properties. Therefore, genetic lineage tracing becomes a complementary approach to study skeletal stem and progenitor cells (SSPCs) *in vivo* (Fig. 2C). The most popular system to perform lineage tracing is Cre-loxP [48], where Cre or CreER (inducible by tamoxifen administration) [49] expression is driven by SSPC-specific promoters. This, in turn, removes the “STOP” sequence flanked by loxP sites to activate downstream reporters. Both Cre-expressing cells and their progenies will be permanently labeled by the reporter, which indicates the differentiation potential of SSPCs *in vivo*. The accuracy of this technique largely depends on the specific expression of Cre recombinase in targeted cells. Unfortunately, this is not always the case. To overcome this caveat, dual recombinase lineage tracing has been developed. It includes two orthogonal recombination systems, namely, Dre-rox and Cre-loxp [50] (Fig. 2C), thereby allowing simultaneous or mutually exclusive tracing of Dre^+^ and/or Cre^+^ cells [50]. This novel technique has been recently adapted to study SSCs at different developmental stages [51].

Although lineage tracing provides critical insights into the cell fate determination of SSPCs *in vivo*, the purity of the initially labeled cells is variable because SSCs cannot be purified by one or two marker genes that drive Cre or Dre expression [52]. Recent studies have combined genetic lineage tracing and single-cell RNA-sequencing (scRNA-seq) to dissect the heterogeneity and functional divergence within SSPCs [41, 53, 54] (Fig. 2D). After identifying putative SSC populations by scRNA-seq, flow cytometry cell sorting coupled with *ex vivo* differentiation or *in vivo* transplantation can be used to test their stem cell activity, which is a key supplementation to lineage tracing studies [41].

## MSCs vs. SSCs

In the 1960s, Friedenstein et al. found that ectopic bones are formed after heterotopic transplantation of bone marrow in diffusion chamber [55], or under the kidney capsule [56]. Within the ectopic bones, blood cells, and vasculatures are derived from the recipient, while the skeletal and stromal cells are derived from the donor, suggesting that hematopoietic and skeletal lineage cells are generated by distinct stem/progenitor cells [56]. In the 1970s, Friedenstein et al. continued to show that *ex vivo* culture of bone marrow cells generates fibroblast colonies, which, after expansion and *in vivo* transplantation, can differentiate into bone and transfer the hematopoietic microenvironment [57, 58]. Therefore, these colony-forming fibroblasts were named “bone marrow osteogenic stem cells” [7] or “stromal stem cells” [8]. In 1991, Caplan proposed the name “mesenchymal stem cells” [9], which was widely used afterwards.

In 2006, the International Society for Cellular Society Therapy proposed three minimal criteria for defining human MSCs: (i) adherent to plastic culture dish; (ii) positive for CD105, CD73, and CD90; and (iii) negative for CD45, CD34, CD14, CD11b, CD79α, CD19, and HLA-DR [59]. However, cells that meet these criteria are heterogeneous fibroblasts/stromal cells that exist in almost all solid tissues, precluding the enrichment of stem cell activity [60]. Furthermore, MSCs isolated from different tissues showed fundamental differences in terms of self-renewal and differentiation capacities, calling into attention that the tissue origin must be specified whenever MSCs are mentioned [61, 62]. The promiscuous use of the term “MSC” has led to a large number of studies with conflicting claims. So even Caplan suggested that the name should be changed [63]. Bone marrow MSCs were first identified, the stem cell activity of which has been stringently proved by clonal analysis *in vitro* and *in vivo* [64, 65]. In contrast, MSCs from other non-skeletal tissues, such as the spleen, muscle, and umbilical cord, were loosely defined by non-clonal and *in vitro* assays and they cannot undergo osteogenesis after *in vivo* transplantation [66–68]. Therefore, SSCs appears to be a more appropriate name for MSCs isolated from the skeletal system to reflect their tissue origin and multilineage differentiation potential [33, 60].

In the following sections, we summarize recent progresses in SSC biology in distinct skeletal compartments: the bone marrow, growth plate, perichondrium, periosteum, and cranial suture.

## Bone marrow SSCs

**Figure 3.**
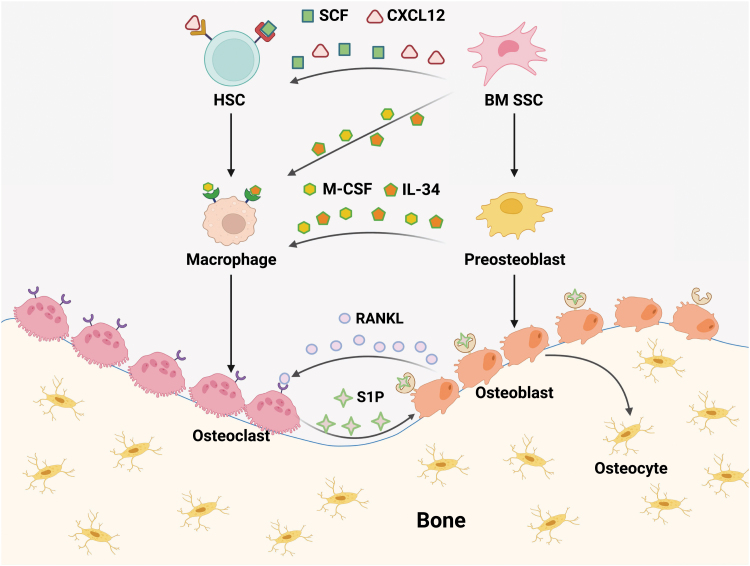
Bone and blood crosstalk at multiple cellular levels. Bone remodeling is orchestrated by the fine balance between osteoblast and osteoclast activities, which are responsible for bone formation and resorption, respectively. Osteoblasts/osteocytes are derived from bone marrow (BM) SSCs, while osteoclasts are derived from macrophages, which are the progenies of HSCs and monocytes. BM SSCs secrete SCF and CXCL12 to maintain HSCs in the perivascular niche [69–71]. BM SSCs and their downstream progenitor cells also secrete M-CSF and IL-34 to regulate the differentiation of macrophages [72, 73]. Osteoblasts secrete RANKL to promote osteoclast differentiation [74–76], while osteoclasts secrete S1P to promote osteoblast differentiation [77, 78]. Therefore, the crosstalk between bone and blood is mediated by HSCs and SSCs, as well as their descendants.

Bone marrow SSCs not only govern osteogenesis in the adult skeleton, but also organize the hematopoietic niche by intimately associating with HSCs in the perivascular region. By orchestrating the skeletal and hematopoietic differentiation hierarchy, bone marrow SSCs and HSCs initiate complex crosstalk between bone and blood at multiple cellular levels (Fig. 3). Notably, several phenotypic markers for prospective isolation of bone marrow SSCs have been discovered in the past two decades. Sacchetti et al. found that CD146 labels perisinusoidal reticular cells that are enriched of CFU-F activity in the human bone marrow [65]. *Ex vivo* cultured CD146^+^ cells form ectopic bones and maintain the hematopoietic microenvironment after subcutaneous transplantation [65]. Interestingly, digestion and culture of the ectopic bones continues to produce CD146^+^ CFU-F colonies, demonstrating their self-renewal capacity [65]. Morikawa et al. showed that Pdgfrα^+^Sca-1^+^CD45^-^Ter119^-^ (PαS) cells enrich a subset of mouse bone marrow MSCs, which exhibit high clonogenic activity, and give rise to hematopoietic niche cells, osteoblasts, and adipocytes following *in vivo* transplantation [79]. Importantly, PαS cells are quiescent and mainly localized in the perivascular region of the bone marrow, reminiscent of CD146^+^ cells in the human bone marrow [79]. Sugiyama et al. found that HSCs are in close contact with CXCL12-abundant reticular (CAR) cells associated with the bone marrow vascularture [80]. CAR cells can differentiate into osteoblasts and adipocytes. Genetic ablation of CAR cells not only reduced osteogenesis and adipogenesis *in vivo*, but also decreased the number of hematopoietic cells in the bone marrow [81]. Méndez-Ferrer et al. found that Nestin-GFP^+^ cells enrich MSC activity in the mouse bone marrow and highly express HSC maintenance factors to organize the hematopoietic niche [82]. Of note, the Nestin-GFP transgene seems to be ectopically expressed in the perivascular stromal cells, in which the expression of endogenous *Nes* gene could not be detected [4, 69]. Therefore, cautions should be taken that Nestin-GFP^+^ cells are not equivalent to Nestin^+^ cells in the adult bone marrow.

LepR^+^ perivascular stromal cells highly express HSC niche factors, such as SCF and CXCL12, to promote HSC maintenance and bone marrow retention, suggesting that they represent a critical component of the HSC niche [69–71]. Zhou and Mizoguchi et al. performed genetic lineage tracing with Lepr-Cre, and found that LepR^+^ cells appear in the periosteal and bone marrow regions around perinatal stage [4, 83]. In adult mice, LepR^+^ cells account for about 0.3% of the whole bone marrow cells and 94% of CFU-F colonies formed *ex vivo* are derived from LepR^+^ cells. Importantly, LepR^+^ cells give rise to most osteoblasts and adipocytes in the adulthood under homeostasis, as well as chondrocytes during fracture healing and cartilage repair [4]. These data strongly suggest that, similar to Nestin-GFP cells and CAR cells [81, 82], LepR^+^ cells enrich bone marrow SSCs and organize a perivascular HSC niche. Yue et al. found that conditional deletion of *Lepr* from limb bones led to increased osteogenesis and decreased adipogenesis, while activation of Lepr signaling in SSCs by high fat diet feeding showed opposite effects [84]. These results suggest that LepR is not only a marker of bone marrow SSCs, but also regulates their cell fate determination by sensing systemic nutritional status [84]. Key transcription factors, such as *Foxc1* and *Ebf3*, were also shown to tightly regulate the differentiation of LepR^+^ SSCs, as well as their hematopoietic niche function [85, 86].

In addition to HSC niche factors, LepR^+^ SSCs also secrete a panel of bone growth factors, such as *Osteolectin*, the level of which significantly decreases with age [87]. *Osteolectin*-deficient mice show accelerated bone loss during aging, while administration of recombinant Osteolectin prevents and reverses osteoporosis by promoting bone formation [87]. Mechanistic study revealed that Osteolectin binds to α11β1 integrins to activate Wnt signaling pathway and osteogenic differentiation in SSCs [88]. Osteolectin inhibits the serine protease activity of Fap, an osteogenic suppressor, thereby promoting osteogenic differentiation by SSCs [89]. Interestingly, parathyroid hormone (PTH) promotes *Osteolectin* expression by bone marrow SSCs, partly mediating the anabolic effect of PTH on bone formation [90]. Osteolectin^+^ cells represent a periarteriolar subset of LepR^+^ cells, regulating osteogenesis, and lymphopoiesis in response to mechanical stimuli [91].

## Heterogeneity within bone marrow SSCs

The development of single-cell technology has enabled us to unbiasedly dissect the heterogeneity within bone marrow SSCs. Tikhonova et al. performed scRNA-seq for Lepr-Cre^+^, VE-Cadherin-Cre^+^, and Col2.3-Cre^+^ cells in mouse adult long bones under steady state and after 5-FU treatment [53]. They subdivided Lepr-Cre^+^ cells into four clusters: Mgp^high^, Lpl^high^ (adipogenic biased), as well as Wif1^high^ and Spp1^high^Ibsp^high^ (osteogenic biased) [53]. Baryawno et al. performed scRNA-seq for non-hematopoietic cells in crushed mouse long bones under homeostatic and leukemic conditions, and divided them into 17 cell clusters including bone marrow, endosteal, and periosteal cells [92]. Unfortunately, only a small number of BMSCs were captured by their analysis, which precludes in-depth resolution of their heterogeneity of bone marrow SSCs [92]. Zhong et al. performed scRNA-seq analysis of Col2-Cre^+^ cells and identified a population of marrow adipogenic lineage precursors (MALPs) that highly express adipocyte markers (e.g. Adipoq). Importantly, these MALPs are different from mature adipocytes that contain lipid droplets [54], reminiscent of LepR^+^/CAR cells. Baccin et al. combined scRNA-seq and spatial transcriptomics (LCM-Seq) to construct a single-cell atlas of both hematopoietic stem/progenitor cells and non-hematopoietic cells in the mouse bone marrow. They subdivided CAR cells into perisinusoidal Adipo-CAR cells that are adipogenic-primed, as well as periarteriolar Osteo-CAR cells that are osteogenic-primed [93]. Leimkuhler et al. revealed two pro-fibrotic clusters originating from LepR^+^ cells, which lose SSC properties during fibrosis and undergo functional reprogramming to acquire a pro-fibrotic and inflammatory phenotype in myeloproliferative neoplasms [94].

Recently, long bone Lepr-Cre^+^ cells under homeostatic and stress conditions were analyzed by scRNA-seq [41]. Mo et al. performed comprehensive analyses of young, aged, rosiglitazone-fed, sublethally irradiated and fractured mice, and subdivided long bone Lepr-Cre^+^ cells into four lineages, including adipogenic, osteogenic, periosteal, and cycling cells [41]. Within the adipogenic lineage, a novel Notch3^+^ subset closely associated with the bone marrow vasculatures was found. This subpopulation is quiescent and expresses the highest level of HSC maintenance factors, suggesting that Notch signaling mediates the interaction between vascular endothelial cells and LepR^+^ SSCs, critical for the maintenance of the perivascular HSC niche [41]. Within the osteogenic lineage, transcription factors Npdc1 and Hoxb2 were shown to promote osteochondrogenic differentiation and fracture healing [41]. Although very few periosteal Lepr-Cre^+^ cells were found under steady state, they quickly expand in response to sublethal irradiation or bone fracture. Importantly, a Sca-1^+^ periosteal subset was identified, which exhibits high clonogenic and adipogenic, but limited osteochondrogenic potential [41]. Therefore, although Sca-1 has been proposed as an MSC marker within the bone marrow compartment [79], it does not apply to periosteal SSCs (p-SSCs). Interestingly, these observations are consistent with a previous study demonstrating that CD45^−^CD31^−^Sca-1^+^ cells enrich adipogenic progenitors in crushed mouse long bones [95], which might include the periosteal Sca-1^+^ cells discovered by Mo et al.

## Growth plate SSCs

The growth plate also contains SSC activity. In long bones, growth plate is located in between the epiphysis and diaphysis shaft, which can be divided into three structurally different zones [17]. The resting zone contains round chondrocytes that are relatively quiescent. The proliferative zone is a columnar arrangement of flat chondrocytes. The hypertrophic zone contains enlarged and non-proliferating chondrocytes. It was previously thought that apoptotic cell death is the terminal fate of hypertrophic chondrocytes, upon which new osteoid is laid down by surrounding osteoblasts to initiate mineralization [96–99]. However, some hypertrophic chondrocytes display asymmetric cell divisions [100], in which one daughter cell assumes an osteogenic fate, while the other ultimately vanishes by apoptosis [100]. Consistent with this, lineage tracing studies show that some hypertrophic chondrocytes do survive and differentiate into osteoblasts, osteocytes, and BMSCs [20–22, 101, 102]. Zhou et al. performed lineage tracing using Col10-Cre and Acan-CreER to demonstrate the transdifferentiation of hypertrophic chondrocytes into osteoblasts during skeletal development, and in bone callus generated during fracture repair [21]. Yang et al. used Col10-CreER to perform temporally controlled lineage tracing of hypertrophic chondrocytes [20]. The Col10-expressing cells labeled at E13.5 lasts to E18.5, P5, and 1 month after birth and generate Osx^+^ pre-osteoblasts, Col1a1^+^ osteoblasts, as well as Sost^+^ osteocytes. When labeled at P5, hypertrophic chondrocytes-derived osteogenic cells can persist into adulthood [20]. Ono et al. used Col2-CreER, Acan-CreER, and Sox9-CreER to perform lineage tracing, and showed that growth plate chondrocytes can contribute to osteoblasts, adipocytes, and BMSCs for more than 1 year [22]. Together, these studies demonstrated that chondrocytes, especially hypertrophic chondrocytes, play critical roles in bone development, maintenance, and regeneration by transdifferentiating into osteolineage cells. However, the detailed transformation process and underlying regulatory mechanism await further elucidation.

In parallel to the above lineage tracing studies, Chan et al. utilized a panel of cell surface markers (CD45^−^Ter119^−^Tie2^−^Thy1^−^6C3^−^AlphaV^+^CD105^-^CD200^+^) to define mouse skeletal stem cells (mSSCs) in the neonatal growth plate [103]. These mSSCs can be prospectively isolated by flow cytometry and serially passaged *ex vivo*. After renal subcapsular transplantation, they form bone, cartilage and stromal cells that support hematopoiesis, but not adipocytes, clearly distinguishing them from bone marrow SSCs. When the ectopic bones were digested, sorted again using the same phenotypic markers and subjected to secondary transplantation, trilineage differentiation is preserved, suggesting that mSSCs self-renew *in vivo* [103]. Importantly, the same research group also identified an analogous hSSC population (PDPN^+^CD146^−^CD73^+^CD164^+^) in the growth plate of 17-week human long bones [104]. Mizuhashi et al. constructed Pthrp-CreER mice to track the cell fate of resting zone chondrocytes [105]. They showed that Pthrp^+^ cells express mSSC markers and generate proliferating and hypertrophy chondrocytes, which further give rise to a portion of osteoblasts and CXCL12^+^ BMSCs [105]. Genetic ablation of Pthrp^+^ cells using the iDTR system affects bone elongation. Therefore, resting zone Pthrp^+^ cells constitute an important part of growth plate SSCs [105]. A population of Pthrp^-^FoxA2^+^ SSCs was also identified on top of the resting zone [106]. Interestingly, they are capable of producing some Pthrp^+^ cells, and exhibit postnatal osteo-chondroprogenitor activity as revealed by *in vivo* lineage tracing [106]. The FoxA2^+^ cells quickly expand in response to growth plate injury and participate in the repair process [106], suggesting a putative upstream population of Pthrp^+^ SSCs in the growth plate.

More recently, Shu et al. explored the transition between growth plate chondrocytes and LepR^+^ bone marrow SSCs during postnatal bone growth [51]. Using a dual recombinase lineage tracing system, they found that osteoblasts before puberty are mainly derived from Acan^+^ chondrocytes in the growth plate, whereas osteoblasts after puberty are mainly derived from LepR^+^ bone marrow SSCs. Growth plate chondrogenesis regulates longitudinal bone growth, while bone marrow SSCs regulates appositional bone growth and bone remodeling in adulthood [51].

## Perichondrial SSCs

During limb bud development, core mesenchymal cells condense and differentiate into the cartilage primordium, while surrounding cells become the perichondrium, which contains osteochondral progenitors that later invade the POC [107]. During postnatal bone growth, perichondrium can be found around the groove of Ranvier, from which osteochondral progenitors can migrate to the articular surface, as well as the resting zone of the growth plate [108].

Lineage tracing studies have identified multiple populations of osteochondral progenitors in the perichondrium. Osx-CreER^+^ cells were detected in the perichondrium of embryonic long bones, which invade the cartilage primordium along with blood vessels and give rise to osteoblasts, osteocytes, and stromal cells in the bone marrow cavity [18, 19]. Similarly, Gli1-CreER^+^ cells were detected in the perichondrium at E13.5. They give rise to osteoblasts, osteocytes, growth plate cartilage, bone marrow adipocytes, and LepR^+^ stromal cells in the adult skeleton [109]. By lineage tracing of Hoxa11-CreER, Pineault et al. found that Hoxa11 labels perichondrial cells and some chondrocytes in the zeugopod region of the developing limb. Hoxa11^+^ cells labeled at E13.5 contribute to postnatal zeugopod skeleton by producing BMSCs, osteoblasts, adipocytes, and periosteal cells for at least a year [110]. However, the expression of Hoxa11 is spatially restricted, as no labeling and contribution were observed in the stylopod region [110].

To study human embryonic skeletogenesis in an unbiased way, He et al. performed scRNA-seq and delineated the transcriptomic landscape of 5-week limb buds, as well as 8-week long bones and calvarial bones in human embryos [40]. Osteo-chondrogenic progenitors (OCPs) highly expressing *TWIST2* and *PDGFRA* were detected in both limb bud and long bone samples. As a subpopulation of OCPs, embryonic skeletal stem/progenitor cells (eSSPCs) enriched of the FOXP1/2 transcriptional regulatory network was discovered in 8-week long bones. eSSPCs are mainly localized in the perichondrium and can migrate into the nascent POC. Phenotypic markers (PDGFRA^low/−^PDPN^+^CADM1^+^) were used to prospectively isolate eSSPCs, followed by functional characterizations *in vitro* and *in vivo* to demonstrate their self-renewal and osteo-chondrogenic capacities. Unlike growth plate and bone marrow SSCs in the postnatal skeleton, eSSPCs do not organize the hematopoietic microenvironment upon renal subcapsular transplantation [40]. Interestingly, perichondrial eSSPCs are evolutionarily conserved in mouse embryonic long bones, with enriched Foxp1/2/4 transcriptional regulatory networks [40]. Their contribution to skeletal development, maintenance, and regeneration needs further investigation.

## p-SSCs

With vasculature invasion and innervation, the perichondrium is gradually transformed into periosteum during bone growth. The periosteum is a thin connective tissue on the outer bone surface and can be separated into two layers [111]. The inner layer near the bone surface is called the cambium layer that contains SSPCs and vasculature, while the outer layer near the skeletal muscle is called the fibrous layer that is composed of fibroblasts and collagen fibers.

Smooth muscle actin (α-SMA) is a marker of pericytes and smooth muscle cells. Interestingly, periosteal αSMA-CreER^+^ cells rapidly proliferate after fracture to give rise to the majority of osteoblasts and chondrocytes in the bone callus [112]. Importantly, p-SSCs are enriched in Mx1^+^αSMA^+^ cells, which differentiate into osteoblasts under homeostasis, and migrate to the site of injury in a CCL5/CCR5-dependent manner to produce osteoblasts and chondrocytes during fracture repair [113]. The heterogeneity within αSMA-CreER^+^ lineage cells was recently resolved by scRNA-seq [114], confirming the existence of SSPCs, as well as fibroblasts and perivascular cells.

Sox9-CreER^+^ cells are distributed in the growth plate, periosteum and endosteum of adult long bones [115], but mainly in the periosteum of rib bones [116]. During early stage of long bone repair, Sox9-CreER^+^ cells are mobilized to the fracture site and differentiate into chondrocytes, osteoblasts and osteocytes [115]. However, the relative contribution of periosteal and endosteal Sox9-CreER^+^ cells remains unclear. Duchamp et al. studied Prx1-Cre^+^ periosteal cells in adult long bones and found that they not only undergo multilineage differentiation *in vitro*, but also promote fracture repair after primary and secondary transplantation *in vivo*, suggesting the existence of self-renewing SSCs in the periosteum [117]. The authors found that *Periostin* is significantly up-regulated and critically regulates p-SSC function in the extracellular matrix after fracture.

Cathepsin K (Ctsk) is a cysteine protease highly expressed in osteoclasts for bone matrix resorption [118]. Interestingly, a population of Ctsk-Cre^+^ periosteal stem cells (PSCs) was identified in both adult long bones and cranial sutures [37]. Ctsk-Cre^+^ cells appear in the perichondrium as early as E14.5, and remain in the periosteum after birth. Using phenotypic markers that identify mSSCs in the bone marrow [103], Debnath et al. prospectively isolated a PSC population within Ctsk-Cre^+^ periosteal cells. These PSCs undergo trilineage differentiation *in vitro*, but only osteogenic differentiation after renal subcapsular transplantation, suggesting that it is responsible for intramembranous ossification under steady state [37]. Remarkably, PSCs shift their cell fate toward endochondral ossification during fracture repair, producing chondrocytes and osteoblasts within the bone callus [37].

## Cranial suture SSCs

**Figure 4.**
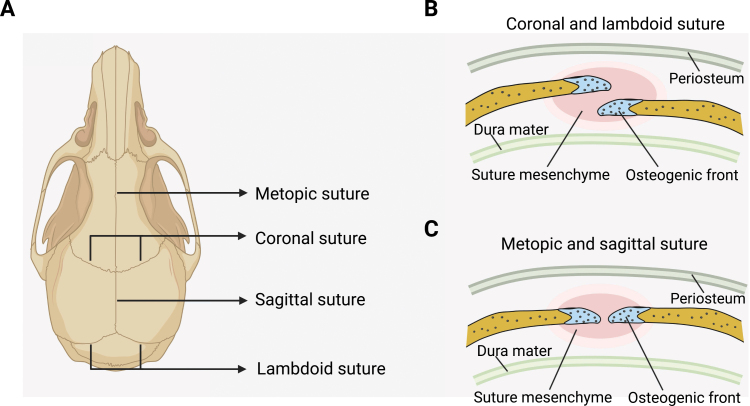
Cranial sutures in the mouse skull. (A) Graphical illustration of the major cranial sutures in the mouse skull. (B) The osteogenic fronts of coronal and lambdoid sutures partially overlap with each other. Blue: osteogenic fronts; Pink: suture mesenchyme. (C) The metopic and sagittal sutures are connected end-to-end.

**Figure 5.**
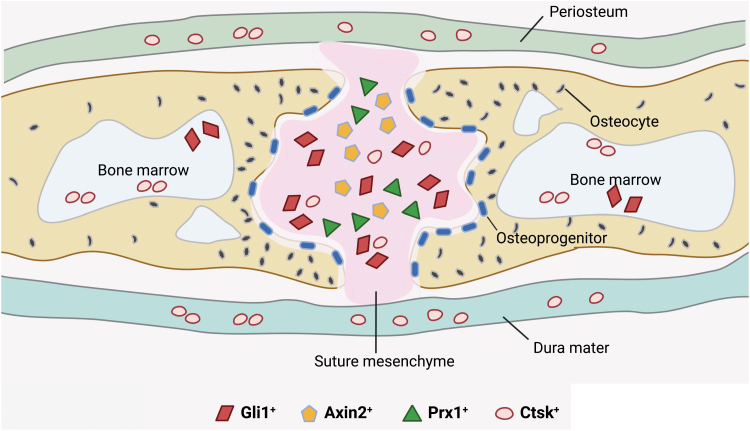
Different populations of SSCs in the cranial suture. Gli1^+^ cells are gradually restricted to the suture mesenchymal 1 month after birth. Axin2^+^ and Prx1^+^ cells are localized in the suture mesenchyme. Ctsk^+^ cells are localized in the suture mesenchyme, periosteum, dura mater, and the marrow cavity of the skull.

Remaining unmineralized to support continuous skull and brain development after birth, the cranial sutures are dense fibrous tissue at the junction of calvarial bones (Fig. 4A). Cranial sutures at different locations have distinct embryonic origins [119]. The sagittal and metopic sutures are derived from neural crest, while the coronal sutures are derived from paraxial mesoderm. The cranial suture consists of osteogenic fronts, mesenchymal cells in between the osteogenic fronts, periosteum, and dura mater. The coronal and lambdoid sutures contain partially overlapping osteogenic fronts, while metopic and sagittal sutures are connected end-to-end (Fig. 4B and 4C) [120]. The suture mesenchyme enriches SSCs and osteoprogenitors that can differentiate into osteoblasts at the osteogenic front to support skull expansion [121] (Fig. 5).

As a key downstream effector of the Hedgehog signaling [122], Gli1 is restrictedly expressed in the suture mesenchyme from 1 month after birth [123]. Gli1^+^ cells undergo trilineage differentiation *in vitro*, and give rise to all craniofacial bones and repair injuries *in vivo*. Genetic ablation of Gli1^+^ cells causes craniosynostosis, suggesting that SSC activity is enriched in this population [123]. Axin2 is a classical Wnt target gene and marks slow-cycling mesenchymal cells in the calvarial sutures [36]. High levels of Gli1 expression in Axin2^+^ cells suggest an overlap between these two cell populations. Axin2^+^ cells self-renew and differentiate *in vitro*, and form cartilage and bone after renal subcapsular transplantation. Axin2^+^ cells also expand in response to calvarial injury, and repair bone defects by generating osteolineage cells [36], indicative of suture SSC activity in these cells. The transcription factor Prx1 is also involved in craniofacial development [124]. Postnatal Prx1^+^ cells are mainly localized in the suture mesenchyme and contribute to the repair of calvarial bone defects [125]. Ctsk-Cre^+^ SuSCs, the counterpart of long bone PSCs, are also located in the cranial suture and exhibit SSC activities [37]. However, the extent to which these SuSCs overlap with Gli1^+^, Axin2^+^, and Prx1^+^ suture SSCs remains to be elucidated.

## Conclusions and future directions

The past two decades have witnessed substantial progresses in the identification and characterization of SSCs from distinct skeletal compartments. We summarize our current knowledge about SSCs in limb bones and craniofacial bones, including bone marrow, growth plate, perichondrial, periosteal, and cranial suture SSCs (Table 1). Nevertheless, there are still critical issues that remain to be addressed by future studies, including, but not limited to, the following directions.

**Table 1.** Lineage tracing studies reveal SSCs from different skeletal compartments

Location	Cre lines	Pulse time	Lineage contribution	References
Bone marrow	Mx1-Cre	pIpC at 6−8 weeks	Osteoblasts	Park et al. [126]
	Osx-CreER	Tam at P5	Osteoblasts, adipocytes, chondrocytes during fracture healing	Mizoguchi et al. [83]
	Lepr-Cre	—	Osteoblasts, adipocytes, chondrocytes during fracture healing	Zhou et al. [4]
	Grem1-CreER	Tam at P1	Osteoblasts, chondrocytes	Worthley et al. [127]
	Ebf3-CreER	Tam at 10 w	Osteoblasts, adipocytes	Seike et al. [86]
	Cxcl12-CreER	Tam at P3, P21 and 8 weeks	Osteoblasts, adipocytes, chondrocytes during fracture healing	Matsushita et al. [128]
Growth plate	Grem1-CreER	Tam at P1	Osteoblasts, growth plate chondrocytes	Worthley et al. [127]
	Pthrp-CreER	Tam at P6	Osteoblasts, chondrocytes, BMSCs	Mizuhashi et al. [105]
	FoxA2-CreER	Tam at P14-18, P28-37	Osteo-chondro-progenitor activity at P14, chondrogenic potential beyond P28	Muruganandan et al. [106]
Perichondrium	Prx1-CreER	Tam at E15.5 and E16.5	—	Kawanami et al. [129]
	Osx-CreER	Tam at E13.5	Osteoblasts, stromal cells	Maes et al. [18] Mizoguchi et al. [83]
	Gli1-CreER	Tam at E13.5	Osteoblasts, adipocytes, chondrocytes, stromal cells	Shi et al. [109]
	Hoxa11-CreER	Tam at E13.5	Osteoblasts, adipocytes, stromal cells, periosteum cells	Pineault et al. [110]
Periosteum	αSMA-CreER	Tam at 5 w	Osteoblasts and chondrocytes in healing callus	Matthews et al. [112]
	Sox9-CreER	Tam at 12 weeks	Osteoblasts and chondrocytes in healing callus	He et al. [115]
	Ctsk-Cre	—	Osteoblasts, chondrocytes in fracture callus	Debnath et al.[37]
Suture mesenchyme	Axin2-CreER	Tam at 4 weeks	Osteoblasts, mesenchymal cells	Maruyama et al. [36]
	Prx1-Cre	—	Osteoblasts, mesenchymal cells, osteogenic fronts, periosteum	Wilk et al. [125]
Suture mesenchyme and bone marrow	Gli1-CreER	Tam at 4 weeks	Sutural cells, osteogenic fronts, periosteum, dura	Zhao et al. [123]
Suture mesenchyme, bone marrow, periosteum, and dura	Ctsk-Cre	—	Osteoblasts, mesenchymal cells, periosteum, dura	Debnath et al. [37]

Methodologies for assessing SSC activity require further optimization. Serial passaging and differentiation *ex vivo*, subcutaneous or renal subcapsular transplantation *in vivo*, as well as genetic lineage tracing and ablation should be combined to fully demonstrate the self-renewal and multilineage differentiation capacities of putative SSCs. Importantly, clonal analysis, both *in vitro* and *in vivo*, indispensable for defining a stem cell population, is unfortunately overlooked in most SSC studies. It should also be noted that *in vitro* differentiation is a relatively artificial assay, and *ex vivo* expansion is a strong stress that could dramatically change SSC properties. Therefore, direct transplantation of cells of interest without *ex vivo* culture is a more reliable approach. Robust transplantation assays that allow quantitative measurement of SSC activities with a low input of primarily isolated cells, analogous to long-term competitive transplantation in the HSC field, would greatly advance SSC biology. Transplantation sites other than the subcutaneous cavity and kidney capsule could be explored for a more permissive mechanical and biochemical microenvironment for SSC to engraft. Finally, cutting-edge technologies, such as scRNA-seq and spatial transcriptomics, should be integrated and continuously applied to SSC biology for discovering novel stem cell populations and regulatory mechanisms.

Many fundamental questions regarding SSCs remain unresolved. For example, although mSSCs, hSSCs, PSCs, and eSSPCs have been discovered, their embryonic origins are still elusive. Whether a subpopulation of limb bud mesenchymal progenitors predisposed to chondrogenic differentiation represents the earliest SSCs is largely unknown. Also, the aging mechanisms of SSCs, including both cell-intrinsic and -extrinsic mechanisms, and their contribution to the progression of skeletal diseases are poorly understood. Whether rejuvenation of SSCs is a feasible way of improving skeletal health needs further investigations. In addition, little is known about SSCs in axial bones, such as the vertebrae, sternum, and rib bones. They may or may not share the same phenotypic markers and properties as SSCs in appendicular or craniofacial bones. Finally, the application of SSCs in regenerative medicine is just emerging, and apparently more efforts should be made to fulfill bench-to-bedside translation. Transplantation of region-specific SSCs with appropriate cell scaffolds, and activation of endogenous SSC activity represent two major avenues for translational research, which may prevent or treat skeletal disorders in the years to come.
